# Systematic errors in the perception of rhythm

**DOI:** 10.3389/fnhum.2022.1009219

**Published:** 2022-11-09

**Authors:** Jiaan Mansuri, Hassan Aleem, Norberto M. Grzywacz

**Affiliations:** ^1^Department of Biology, Georgetown University, Washington, DC, United States; ^2^Interdisciplinary Program in Neuroscience, Georgetown University, Washington, DC, United States; ^3^Department of Molecular Pharmacology and Neuroscience, Loyola University Chicago, Chicago, IL, United States; ^4^Department of Physics, Georgetown University, Washington, DC, United States; ^5^Department of Neuroscience, Georgetown University, Washington, DC, United States; ^6^Department of Psychology, Loyola University Chicago, Chicago, IL, United States

**Keywords:** loss function, Bayesian theory, systematic error, neural noise, temporal prediction, rhythm

## Abstract

One hypothesis for why humans enjoy musical rhythms relates to their prediction of when each beat should occur. The ability to predict the timing of an event is important from an evolutionary perspective. Therefore, our brains have evolved internal mechanisms for processing the progression of time. However, due to inherent noise in neural signals, this prediction is not always accurate. Theoretical considerations of optimal estimates suggest the occurrence of certain systematic errors made by the brain when estimating the timing of beats in rhythms. Here, we tested psychophysically whether these systematic errors exist and if so, how they depend on stimulus parameters. Our experimental data revealed two main types of systematic errors. First, observers perceived the time of the last beat of a rhythmic pattern as happening earlier than actual when the inter-beat interval was short. Second, the perceived time of the last beat was later than the actual when the inter-beat interval was long. The magnitude of these systematic errors fell as the number of beats increased. However, with many beats, the errors due to long inter-beat intervals became more apparent. We propose a Bayesian model for these systematic errors. The model fits these data well, allowing us to offer possible explanations for how these errors occurred. For instance, neural processes possibly contributing to the errors include noisy and temporally asymmetric impulse responses, priors preferring certain time intervals, and better-early-than-late loss functions. We finish this article with brief discussions of both the implications of systematic errors for the appreciation of rhythm and the possible compensation by the brain’s motor system during a musical performance.

## Introduction

Rhythm is a key component of music, possibly preceding melody in its origins (Montagu, 2017). Across the globe, rhythm is used in spiritual rituals and cultural celebrations, increasing social cohesion through group synchronization (Konvalinka et al., 2011; Mogan et al., 2017; Jackson et al., 2018). Furthermore, rhythm is necessary for understanding and producing language (Nazzi et al., 1998; Ramus et al., 1999; Kohler, 2009). Another hypothesis for why our brains like musical rhythm relates to the prediction of time. In the natural world, such prediction may be crucial for survival. For example, animals must predict when to initiate movement to evade a predatory attack, using the so-called margin of error (Kramer and Bonenfant, 1997; Choi and Kim, 2010). This prediction can be aided by observing natural rhythms in movement (Cooper and Blumstein, 2015). Natural selection thus developed dedicated neural circuitry for the prediction of time events and thus, processing temporal regularities such as rhythm.

The cortical and subcortical circuitries of temporal prediction and rhythm processing are fairly distributed in the brain (Karmarkar and Buonomano, 2007; Bengtsson et al., 2009; Grahn, 2012; Sadibolova et al., 2021). These circuitries include areas such as the supplementary motor area, cerebellum, inferior parietal cortex, thalamus, and the basal ganglia (Hardy and Buonomano, 2016; Kotz et al., 2018). These areas allow the processing of rhythm in the brain to be fast and spontaneous (Nozaradan, 2014; Wilson and Cook, 2016). Consequently, our brains are naturally predisposed to process rhythm and this processing is efficient, or fluent. According to the Processing Fluency Theory, such fluent processing should lead to hedonic value attached to rhythm, making it enjoyable (Reber et al., 2004; Aleem et al., 2017, 2019). Further research is needed to understand whether certain properties of rhythms, such as predictability, or complexity, make it less or more enjoyable. Understanding how our brains process rhythms and make temporal predictions may help us answer this question.

Although the brain has evolved relatively accurate mechanisms for processing the progression of time, making predictions about event timing is not easy. The brain only has information about the timing of an event in the ensuing spike trains. Because of neural noise (Ferster, 1996; Miller and Troyer, 2002), the brain must consider an inherently imprecise neuronal spike train triggered after the stimulus (Teich and Khanna, 1985; Teich, 1989; Rokem et al., 2006). Moreover, the prediction must account for the asymmetry inherent in the probability distribution of post-stimulus spike-train timings (De Boer and De Jongh, 1978; Recio-Spinoso et al., 2005; Smith and Lewicki, 2006). After a delay following the stimulus, the probability of a spike rises rapidly and decays slowly. Hence, the time of the stimulus is an estimation processed under complex spiking conditions.

Despite knowing that making predictions about event timing is difficult, we still do not know in detail what type of errors the brain makes. The work of Di Luca and Rhodes (2016) has provided evidence that the brain makes systematic (time-order) errors (Hellström, 1985). Under some conditions, stimuli presented earlier than expected are perceptually delayed, but those appearing on time and later are perceptually accelerated. Di Luca and Rhodes have also proposed a dynamic Bayesian inferential process to explain these results, using the temporal asymmetry described above. In these authors’ proposed process, timing expectations are dynamically updated and thus, the brain’s inference effectively becomes a Bayesian Kalman filter (Barraza and Grzywacz, 2008). However, Di Luca and Rhodes’s range of stimulus parameters was relatively limited. Furthermore, the authors did not explore some important features of Bayesian models. For example, such models often rely on loss functions, which measure the weighted cost of errors. Within the family of these functions, the Better-Early-than-Late losses (BELL) can be employed as a safety margin for predatory evasion (Kramer and Bonenfant, 1997; Choi and Kim, 2010; Cooper and Blumstein, 2015). Simply put, the brain is better off erring towards early predictions of timing onset to escape danger.

The current study proposes and tests a theoretical framework through which the brain predicts beat times in the patterned stimuli underlying musical rhythm. This framework uses Bayesian estimation. Hence, it considers the inherent asymmetric probability distributions of the post-stimulus spike trains, BELL functions, and realistic prior distributions. The framework also attempts to predict different types of systematic errors when estimating the timing of beats in rhythms. We test these predictions psychophysically, probing how the number of beats and interstimulus intervals in rhythmic stimuli modulate these systematic errors. Finally, we develop a computational model based on the Bayesian framework and determine its qualitative and quantitative fits to our data.

### Theory part 1: concepts and predictions

In this section, we outline a theoretical framework for how the brain predicts beat times. We have two purposes here: first, we explain the theoretical concepts and derive experimental predictions. We wait to develop the equations until after the experimental testing because it helps us prune out a variety of possibilities. Second, this section can help the reader understand the theoretical material even if the mathematical developments may be difficult to understand. We thus postpone these developments until Section “Theory part 2: computational modeling” below.

We begin with our definition of beat because our use of the term may be slightly different from how other people use it. We define beat as an individual pulse of sound. This definition is useful for us because such a pulse is what results in a neuronal impulse response in the auditory nerve (De Boer and De Jongh, 1978; Teich and Khanna, 1985; Heil and Peterson, 2015). However, the underlying pulse may extend over a single beat in music. In our experiments, such extension does not occur, so beats and pulses are the same.

**Figure 1:**
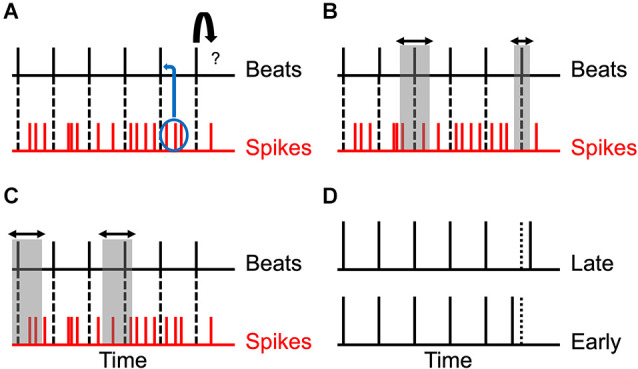
Theoretical considerations, predictions, and test. **(A)** Relationship between beats and spikes. The beats are represented by vertical black lines, while the spikes following each beat are represented by vertical red lines. The problem is to predict the next beat (curved arrow). Each beat produces a different train of spikes and so, the timing of beats must be determined probabilistically from the spikes (blue ellipse and curved arrow). **(B)** Reduction of uncertainty. In an optimal probabilistic estimation, the uncertainty in the timing of the beats decreases as the rhythm progresses (horizontal arrows and vertical gray rectangles). **(C)** Systematic error. Various factors described in the text contribute to systematic errors in the determination of the times of the beats. Thus, the estimated time may be too early or too late (horizontal arrows and vertical gray rectangles). **(D)** Test of the Predictions. To test these predictions, we delivered to subjects isochronic stimuli with varying number of beats and inter-beat intervals. However, the last beat was delivered too early or too late and the task of the subject was to guess which was the case.

The main challenge in determining the time of a beat is that it produces noisy activity in the brain (Figure 1A). Two identical beats do not typically produce the same patterns of spikes in neurons. The brain must use these noisy spike patterns to estimate the time and thus, cannot know for sure when the beat has occurred. The best that the brain can aim for is an optimal probabilistic estimation of the time. We take this optimal approach in our modeling here. Because the estimation is probabilistic, the optimum is Bayesian (Koop, 2003; Knill and Pouget, 2004; Doya et al., 2007; Robert, 2007). In our case, because a rhythm is a repetitive pattern, the optimal process uses the repetitions of the patterns to improve the estimations of the times of the beats (Figure 1B). We can then predict that if the brain uses this optimal approach, the estimation of times of beats improves the longer the rhythm lasts.

However, we have reasons to believe that the errors that the brain makes in the determination of time may be systematic not just random. Thus, people may perceive beats systematically before or after they occur (Figure 1C). One reason to believe in systematic errors is that impulses (for example, beats) tend to produce responses that are temporally asymmetric. They rise fast and fall slowly (De Boer and De Jongh, 1978; Recio-Spinoso et al., 2005; Smith and Lewicki, 2006). Therefore, the majority spikes happen after the peak of this rise-and-fall process. What is the time of the beat given these spikes? To simplify the answer, let us imagine that the beat produces just one spike with a given asymmetric probability distribution. What is the best interval to subtract from the time of this spike to calculate an accurate estimate for the time of the beat? The answer depends on the types of errors that we are willing to make, something characterized by the so-called loss function. For example, if we want to minimize the square of the error (a quadratic loss), then one can prove that this estimation should subtract the average time of the probability distribution. However, because the spike happens after this average most of the time, the correction will tend to lead to a systematic error, predicting a time later than in reality.

Another reason to think that the errors the brain makes may be systematic is that prior beliefs about a rhythmic pattern may have a bias towards certain values (De Jong et al., 2021). As mentioned in the *Introduction*, in the English spoken language, people tend to articulate approximately three to five syllables per second (London, 2012; Patel and Iversen, 2014). Hence, humans are often exposed to rhythms with time intervals of roughly 200 or 300 ms. Consequently, imagine that one is hearing a rhythm whose intervals are shorter than 200 ms. Thus, if one is doing optimal estimation, one would tend to predict the time interval as longer than it really is. Hence, one would estimate the time of the next beat as later than it occurs. The opposite would happen if the rhythm were slow.

A final reason to believe that the brain may make systematic errors is what we call the Better-Early-than-Late loss (BELL). In nature, animals are better off underestimating time than overestimating it to have a margin of error (Kramer and Bonenfant, 1997; Choi and Kim, 2010; Cooper and Blumstein, 2015). More specifically, they will be unable to escape their predators by overestimating their arrival time. With BELL, an optimal estimator would tend to estimate the next beat earlier than it occurs.

In summary, these theoretical considerations lead to two predictions: first, the estimation of times of beats improves the longer the rhythm lasts. Second, the estimation errors may well be systematic, being positive or negative depending on both the duration and frequency of the rhythm. To test these predictions, we ran the following psychophysical experiment (Figure 1D). Subjects heard isochronic rhythms whose variables were the number of beats and the inter-beat interval. The last beat was either later or earlier than expected. The observer’s task was to choose whether the last beat was too late or too early in a two-alternative forced choice. A systematic error would be observed by the subject choosing early or late more than 50% of the time.

## Experimental Methods

### Participants

Seven neurologically healthy participants with normal hearing took part in the experiment (three females, four males, ages 16–64). Each participant took part in eight sessions of approximately 1 h to complete the experiment. Each session was on a different day, with participants completing all sessions in at most 10 days. Two of the participants (Subjects 1 and 2) performed the experiment *in situ* in the lab. The other five participants performed the experiment after downloading the computer code to their own computers. A concern with having five participants use their own computer was that this could add variability to the data, swamping the effects of interest. However, these effects were still evident in the experiment, easing the concern.

Having a large enough number of participants is important for group-statistical inference (Button et al., 2013; Stanley et al., 2018). We thus determined the size of the participant cohort with a power analysis of pilot experiments. Cohen’s h is an appropriate measure of effect size between proportions (Cohen, 2013). The smallest effect was the difference between the percentage of correct responses for a large number of beats and slow rhythms. In this condition, there were 108 observations per participant. To ensure the appropriateness of our sample size, we calculated the smallest possible h value that we could obtain assuming *α* = 0.05 and at 0.9 power. With a sample size of seven participants, the smallest possible h value was 0.09, which was smaller than what was considered a “small” effect size (though see Correll et al., 2020). Therefore, we believed that seven participants was a proper sample size to detect an effect. This sample was smaller than that used by Di Luca and Rhodes (2016), who had 15 participants for a similar experiment. However, we did not need as many participants because we used experimental parameters that were more extreme, magnifying the effects.

Five of the seven participants were naïve as to the purpose of the experiment. However, knowledge of this purpose had no influence on experimental results for the other two participants given that they could not know *a priori* whether their responses were correct.

This experiment was approved by the Georgetown University Institutional Review Board, with all participants giving their consent to participate before engaging in the trials.

### Apparatus and stimuli

We programmed the experiment using the Builder view of PsychoPy3 version 2020.1.0 (Peirce et al., 2019). The experimental stimuli followed the protocol described in Figure 1D. Beat-pattern stimuli were prepared using a standalone version of MATLAB_R2019b (MathWorks Inc., Natick, MA). As is standard in auditory beat experiments, a kickdrum was sampled as the individual beat to be used for each pattern (Treder et al., 2014). We held the duration of the kickdrum waveform constant at 22 ms. Similarly, output volume was maximized at 100% and a constant sampling rate of 44,100 Hz was used as a standard to produce individual beats and beat patterns. We synthesized beat patterns with varying values for the parameters: number of beats in a pattern (N), inter-beat interval before the last beat (Delta), and the relative deviation of the last interval (rho), defined as a fraction change from Delta. Table 1 shows the values tested of N, Delta, and rho for each subject.

**Table 1:** Experimental parameters for each subject.

Subject #	N	Delta (s)	rho
1	(3,4,6,9)	(0.15,0.3,0.6,0.9,1.2)	(−0.15,−0.125,−0.1,−0.075,−0.05,0,0.05,0.075,0.1,0.125,0.15)
2	(3,4,6,9)	(0.15,0.3,0.6,0.9,1.2)	(−0.15,−0.1,−0.05,0,0.05,0.1,0.15)
3	(3,4,6,9)	(0.15,0.3,0.6,1.2)	(−0.15,−0.1,−0.05,0,0.05,0.1,0.15)
4	(3,4,6,9)	(0.15,0.3,0.6,1.2)	(−0.15,−0.1,−0.05,0,0.05,0.1,0.15)
5	(3,4,6,9)	(0.15,0.3,0.6,1.2)	(−0.15,−0.1,−0.05,0,0.05,0.1,0.15)
6	(3,4,6,9)	(0.15,0.3,0.6,1.2)	(−0.15,−0.1,−0.05,0,0.05,0.1,0.15)
7	(3,4,6,9)	(0.15,0.3,0.6,1.2)	(−0.15,−0.1,−0.05,0,0.05,0.1,0.15)

For the experiment to work, the persons performing the experiment on their own devices had to use a computer, not a smartphone or tablet.

### Procedure

Beat pattern stimuli were grouped such that on each day, the effect of varied Delta and rho parameters were tested while N was held constant. We tested each value of N over 2 days, allowing for at least 18 repetitions of each stimulus, a unique beat pattern combination of N, Delta, and rho. Although the duration of experimental sessions varied based on subjects’ response times, we optimized the experiment to decrease the average session to 60 min or less. With shorter times, we hoped to minimize the effects of subject fatigue. Similarly, we held N constant for each session to decrease difficulty without influencing the subjects’ perceptual capacity. Finally, we ordered session days such that the number of beats tested would descend in each consecutive series of sessions. This order allowed subjects to progress from easier perceptual tasks to harder ones. In pilot experiments, we noticed that more beats led to a clearer representation of the tempo, making the task easier. Hence, going from larger to smaller number of beats would facilitate learning, a phenomenon called “Transfer Along a Continuum” or “Easy-to-Hard Effect” (Ahissar and Hochstein, 2004; Liu et al., 2008; Mackintosh, 2009). Therefore, this order of the number of beats made later sessions clearer. However, a concern was whether the descending order of N would confound the behavioral data. Nevertheless, if anything, the order was compatible with the tested hypothesis. If we found an improvement in performance with increasing N, that would be despite the tests making the task with a small N easier.

Beat patterns appeared in random order for each session of N. We tested each pattern in three-part blocks corresponding to a preparatory “ready?” screen, the presentation of the auditory stimulus, and a feedback prompt. After listening to a beat pattern, subjects were prompted by the feedback screen to indicate whether the final beat was heard before or after it was expected to occur (Figure 1D). Before starting the test sessions, subjects heard an exaggerated example of a beat pattern with a rho value increasing or decreasing the final interval by 20%. With this exaggerated trial, we ensured an understanding of the timing differences tested by each trial. The same exaggerated stimuli with N = 5 and Delta = 0.2 s and 1.5 s were used for each trial day throughout the experiment. Subjects, then, underwent a three-part practice session, to understand the given stimuli and task. Successive levels of the practice session increased in difficulty up to that of the trial values of rho used in the test trial. For each test session of N, the practice utilized the same parameters for Delta. We increased or decreased rhos by 30%, 20%, and 15% of the practiced Deltas in the first, second, and third practice levels respectively. Practice trials were not meant to exceed 15 min of the anticipated 60-min average session time.

### Data analysis

We determined the probability of a subject’s ability to predict the occurrence of an early or late final beat in an experimental condition from the responses. Error bars for the probabilities were calculated based on the standard error of the binomial distribution (Krishnamoorthy, 2006). To probe whether two sets of parameters yielded different probabilities, we used two-sided exact binomial tests (Krishnamoorthy, 2006), reporting effect size with Cohen’s h (Cohen, 2013). We interpreted h with Cohen’s rule-of-thumb descriptions. According to him, we had small, medium, and large effect sizes if 0.2 ≤ *h* < 0.5, 0.5 ≤ *h* < 0.8, and *h* > 0.8 respectively. The same two-sided exact binomial tests and Cohen’s h were also helpful when probing trends as a function of a variable like N or Delta. Next, we tested for trends by grouping separately the responses in the lower and larger values of the variable. Finally, for beat patterns for which rho = 0, we calculated the difference between the numbers of “Late” and “Early” responses parametric on N and Delta. Consequently, we could test for trends as described above in this paragraph.

The article also provides statistical evaluations of the group result, by presenting both statistically robust median results and individual data. Such a separation is possible because the results do not come from an overly large number of subjects. Moreover, we use the population-prevalence approach to quantify how common each effect is (Ince et al., 2022). However, we avoided the maximum-a-posteriori estimate of prevalence because our number of subjects was too small for such a technique. We used instead the frequentist approach (Allefeld et al., 2016; Donhauser et al., 2018). To apply it, the prevalence is computed as a percentage, with the standard error estimated from the binomial distribution (Krishnamoorthy, 2006).

## Experimental Results

### Full results for a single subject

**Figure 2:**
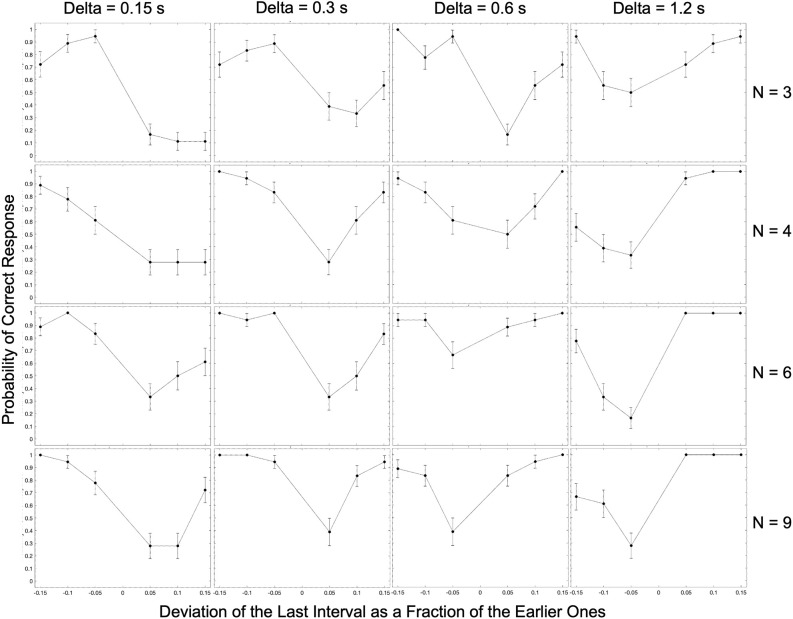
Complete experimental results for a typical observer (Subject 3). The experimental condition in each panel is indicated by the number of beats (N) and the inter-beat interval (Delta). In most panels, the performance exhibits a U-shape behavior as a function of the relative deviation of the last interval. For low inter-beat intervals, subjects too often responded “Early” even when the last beat came late. This systematic error reversed direction at long inter-beat intervals.

In our theoretical considerations, we predicted the occurrence of systematic errors in the perception of beat timing in patterned stimuli. One reason for these predictions was the asymmetrical probability distribution of impulse responses to one beat. Given this asymmetry, subjects would perceive last beats as occurring earlier than the actual stimuli more often. In contrast, if the brain used a Better-Early-than-Late loss (BELL) function, we would tend to predict beats as earlier than the actual stimuli and thus perceive them as late. Similarly, the incidence of perceived early and late responses could be influenced by the brain’s priors about the world. Finally, we predicted that subjects’ estimates of beat timing should become more accurate with more information about the pattern. This would happen, for example, with a large number of beats. We tested these predictions as a function of the number of beats, inter-beat interval, and the relative deviation of the final interval. Figure 2 provides the complete results of this test for one subject.

The most apparent result in Figure 2 is that as the magnitude of the relative deviation of the final interval increases, performance tends to improve. Because our graphs separate negative and positive deviations, this improvement appears as a U-shaped trend. This U-shape is not surprising since with larger deviations, the task gets easier. However, the graphs do reveal two surprising results. First, for shorter inter-beat intervals, we see an asymmetry in the probability of correct responses, wherein predictive performance decreases as the deviation of the final interval shifts from negative to positive values. This asymmetry is statistically significant and has a Cohen’s large effect size when the final deviation is 5% for all conditions with inter-beat intervals of 0.15 and 0.3 s (two-sided exact binomial test, *n* = 188, *p* < 2 × 10^−8^, Cohen’s *h* = 1.1). In addition, the asymmetry is both significant and large effect size for the inter-beat interval of 0.6 s when we have only three beats (*n* = 24, *p* < 0.007, Cohen’s *h* = 1.9). Second, we also demonstrate the reversal in this asymmetry for longer inter-beat intervals. This reversal is always statistically significant and large effect size when this interval is 1.2 s (*n* = 102, *p* < 2 × 10^−5^, Cohen’s *h* = −1.6). Therefore, the asymmetry depends differentially on the number of beats and inter-beat interval.

In summary, these results suggest that at least some subjects make systematic errors when predicting the timing of beats in patterned stimuli.

### Median results over the population

**Figure 3:**
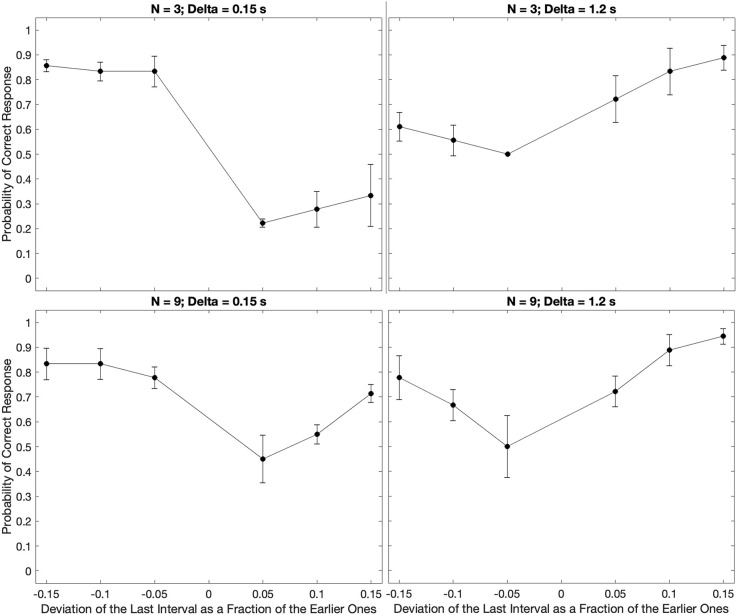
Examples of the median of the data across subjects parametric on the number of beats and the inter-beat interval. Plot conventions are the same as in Figure 2. These plots extend the conclusions from the subject in Figure 2 to the population. We observe both the U-shape behavior as a function of the relative deviations of the last interval and the same systematic errors for low and high inter-beat intervals.

The results presented in Figure 2 are isolated to a single individual. How typical are the early-vs.-late biases for negative and positive relative deviations of the final inter-beat interval? To answer this question, we measured these biases over the population of tested subjects, utilizing median response data across trials to avoid outliers. For simplicity, Figure 3 shows these medians for only the smallest and largest number beats, and the shortest and longest inter-beat intervals.

We find similar but not identical results to the subject in Figure 2 in our population estimate (Figure 3). Indeed, our central estimate confirms the unsurprising U-shaped behavior, as the deviation from the inter-beat interval increased in magnitude. Furthermore, we observe asymmetries in the probability of correct responses. The bias towards perceiving earlier-than-expected beats occurs most when subjects are presented with fewer beats and shorter inter-beat intervals. The asymmetry reverses for the population as the number of beats and inter-beat interval increases. However, some important differences between Figure 2 and Figure 3 are apparent. For example, the asymmetries are less pronounced in the population for a large number of beats. This is indicative of some individuality in the systematic errors, something that we will address later in this section.

These results, thereby, suggest that the trends indicative of systematic error are present in all or most of the population.

### Dependence on the number of beats

**Figure 4:**
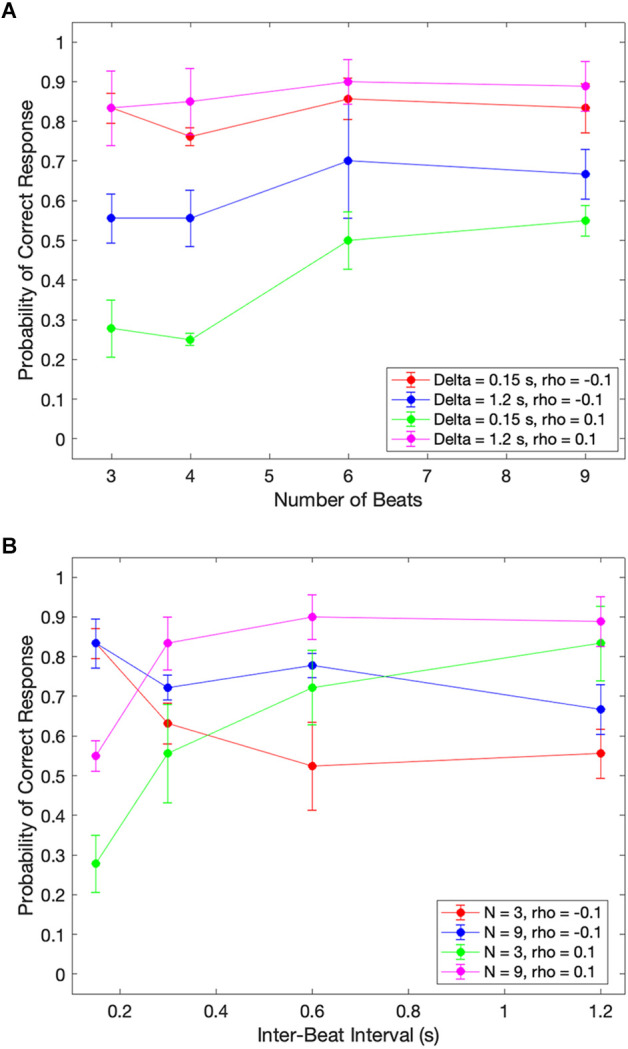
Dependence of median performance as a function of number of beats and the inter-beat interval. **(A)** Dependence on the number of beats parametric on the inter-beat interval and the relative deviation of the last interval. **(B)** Dependence on the inter-beat interval parametric on the number of beats and the deviation of the last interval. The performance as a function of the number of beats is constant except when the inter-beat interval is short and the deviation of the last interval is positive. Thus, performance improves with the number of beats. The performance as a function of the inter-beat interval improves when the deviation of the last interval is positive. However, the opposite happens when this deviation is negative.

In our theoretical considerations, we predicted that increasing the number of beats presented to subjects would improve their estimation of the beat timing. Our argument for this improvement was that the larger number of beats would increase the amount of information. Given the systematic biases presented in Figure 2 and Figure 3, we identify such improvements as fewer asymmetric early-vs.-late biases. We test these predictions with the median across subjects in Figure 4A.

Figure 4A shows that our theoretical prediction for the dependence on the number of beats holds. As this number increases, the probability of a subject responding correctly rises. In the figure, we see this rise by the curves with 0.15 s inter-beat interval getting closer together. The difference between these curves is statistically significantly larger and has a Cohen’s small effect size for the combined 3-and-4-beats data than for the combined 6-and-9-beats results (two-sided exact binomial test, *n* = 680, *p* < 0.002, Cohen’s *h* = 0.3). However, despite getting closer together, the curves do not converge, and a significant asymmetry remains. This remaining asymmetry is statistically significant and has a medium effect size with 9 beats (*n* = 196, *p* < 0.004 for 0.15 s, Cohen’s *h* = 0.6). Although Figure 4A only illustrates the remaining asymmetry and the improvement with the number of beats for rho = 0.1, the result is more general. It also holds for rho = 0.05 and rho = 0.15.

In sum, a larger number of beats helps to improve the performance, but does not eliminate the systematic errors in the perception of beat time.

### Dependence on the inter-beat interval

Our theoretical considerations also predicted the effects of the inter-beat interval on the likelihood of correctly determining the sign of the last-beat deviation. Beat patterns with short inter-beat intervals should more frequently lead to longer than true predicted intervals, eliciting early responses. The opposite would happen for patterns with long inter-beat intervals. A mechanism for how this dependence of systematic errors on inter-beat interval would happen is the prior distribution. A prior peaking in the middle of the experimental range of inter-beat intervals would lead to a longer-than-actual interval estimate for short experimental intervals and the opposite for long intervals. Figure 4B shows the test of these predictions with the median data across subjects.

Figure 4B demonstrates the differential effects of early vs. late deviations on subjects’ probability of responding correctly. For beats that arrived later than expected, subjects’ performance improved as the length of the inter-beat interval increased. This upward trend was statistically significant and had a Cohen’s h medium size effect when probed by grouping separately the responses to inter-beat intervals of 0.15 s and 0.3 s, and 0.6 s and 1.2 s. The probe of the upper trend was the binomial exact test (*n* = 333, *p* < 4 × 10^−8^, Cohen’s *h* = 0.76 for 3 beats and *n* = 444, *p* < 2 × 10^−5^, Cohen’s *h* = 0.53 for 9 beats). In contrast, subjects’ probability of correctly determining an early beat fell as the inter-beat interval rose. This fall was statistically significant for three beats and had a Cohen’s h small size effect (*n* = 355, *p* < 0.005, Cohen’s *h* = −0.4). Again, although Figure 4B only illustrates this fall for rho = −0.1 and the rise for rho = 0.1, the result is more general. The rise also holds for rho = 0.05 and rho = 0.15. However, although the fall is significant for rho = −0.05, the same does not happen for −0.15.

These results highlight the strong effects of the inter-beat interval on systematic errors in the estimation of beat timing.

### Biases as the roots of the systematic errors

**Figure 5:**
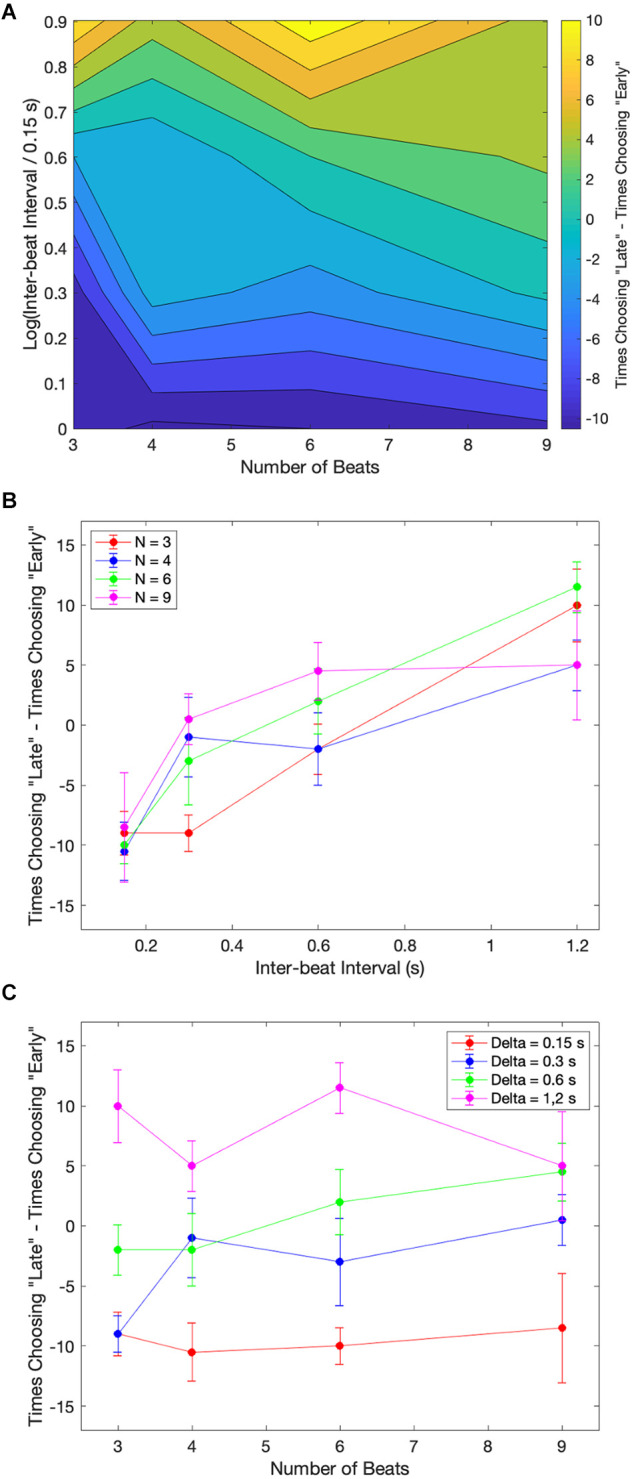
Examples of the median of the data across subjects for the case where the last beat had no deviation. **(A)** Contour plot of the excess of “late” responses over “early” ones. **(B)** Excess of “Late” responses as a function of the inter-beat interval parametric on the number of beats. **(C)** Excess of “Early” responses as a function of the number of beats parametric on the inter-beat interval. Subjects show a bias towards “Early” responses for low inter-beat intervals and the opposite bias for longer ones.

What is the cause of the systematic errors observed in the results so far? A simple hypothesis is that the brain has biases during perceptual processing which manifest as early or late estimates of true event timing. We observe these errors as an outcome. To test this hypothesis, we randomly presented subjects with beat patterns that contained no intervals with relative deviations. Subjects indicated whether they perceived the final beat as occurring before or after it should occur. Figure 5 highlights the outcome of this measurement in terms of the median across the population.

Figure 5A utilizes a contour plot to indicate the trends of the data. Subjects’ bias for each combination of the number of beats and the inter-beat interval is presented in this figure as the subtraction of the number of times the subjects chose late or early. Figure 5A demonstrates that subjects were more likely to shift their biases from early to late as the inter-beat interval increased. This shift is apparent in the change of color from blue to yellow as we move upward in the plot. Figure 5B summarizes this shift. The upward trend in the shift was statistically significant, with Kendall *τ* = 0.625, 0.447, 0.408, and 0.404 in order of increasing number of beats. The probabilities that these coefficients were zero by chance were *p* < 0.0002, 0.007, 0.02, and 0.02 respectively. In contrast, these biases were not changed significantly with the number of beats. None of the Kendall *τ* for the corresponding curves (Figure 5C) were significantly different from zero.

In summary, these results suggest that the early-vs.-late systematic errors are due to biases depending mostly on the inter-beat interval.

### Individuality in the biases

**Figure 6:**
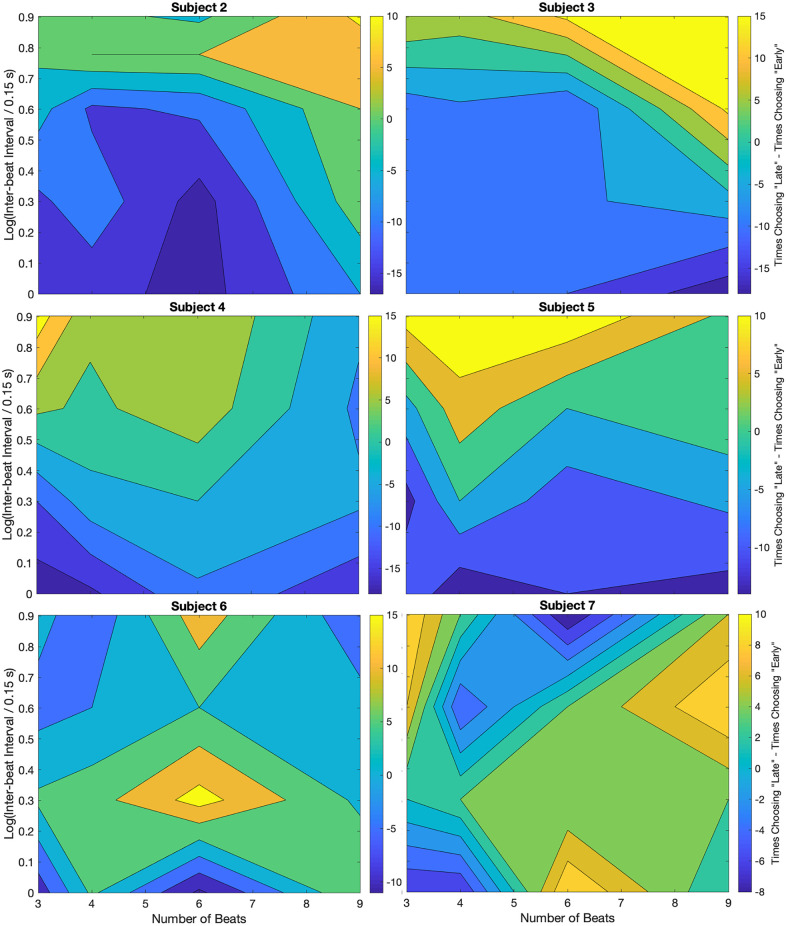
Individuality demonstrated through the non-zero-deviation data for different subjects. Plot conventions are the same as in Figure 5A. As in that figure, most subjects (2–6) show a bias towards “Early” responses for low inter-beat intervals. Similarly, at larger inter-beat intervals, the bias reverses. However, whereas for some subjects the peak reversal happens at high inter-beat intervals and a large number of beats (Subjects 2 and 3), for others the peak is either at a low number of beats (Subjects 4 and 5) or at intermediate inter-beat intervals (Subject 6). Only one subject showed no bias reversal with the inter-beat interval. We discuss possible reasons for this difference between this subject and others in the text.

When comparing Figure 2 with Figure 3, we pointed out that their differences indicated a degree of individuality in the systematic errors. The purpose of this section is to inspect this individuality. We use the results of Figure 5, which suggested that the systematic errors are due to biases in the estimation of the beat time. To study the individuality of these perceptual biases, we present contour plots for each subject participating in trials without relative deviations. Figure 6 shows these contour plots.

For most subjects, we observe the general trend that an increase in the inter-beat interval duration causes a shift from early to late timing estimates. The results of every individual, except for Subject 7, demonstrate that the smallest inter-beat interval tested yields the greatest number of early biases (population prevalence = 86% ± 13%). In our figures, this demonstration is delineated by the darkest blue regions being at the bottom of the plot, where they were always below the yellow. This relative relationship on the plot is indicative of a shift in early-vs.-late bias. The greatest difference between subjects was where in the plot the yellow region laid above the darkest blue. For some subjects, the peak reversal happened at high inter-beat intervals and large number of beats (Subjects 2 and 3). For others, the peak was either at low number of beats (Subjects 4 and 5) or at intermediate inter-beat intervals (Subject 6). Only Subject 7 showed no bias reversal with the inter-beat interval (population prevalence of bias reversal = 86% ± 13%). Post-experiment exit interviews revealed a likely cause for the discrepancy in this subject’s data. Unlike the others, Subject 7 would listen to the pattern, and would try to replicate it with tapping once it was finished being played. So, this subject responded to the short-term memory of the stimuli rather than directly to them.

These results suggested an individuality in terms of the details of the shift in early-vs.-late biases, but not in its gross features.

Could we explain this individuality by the instability of the subjects? Because every data point was measured over two days, the instability of the results could be assessed. We used data like those in Figure 6 for this assessment. For every subject, the correlation coefficients of the data in two consecutive days were always significantly larger than the coefficients across subjects. Consequently, individuality was not due to the subjects’ instability.

### Theory part 2: computational modeling

As explained in Section “Theory part 1: concepts and predictions,” we expect systematic errors in the perception of the timing of beats. The experimental results have confirmed these expectations, revealing complex behavior as a function of the number of beats and the inter-beat interval (Figures 2–6). We now test whether the ideas in Section “Theory part 1: concepts and predictions” can account for these complexities. The goal of this test is not so much to fit the data exactly but to capture their main qualitative points. We thus develop a quantitative theory with realistic elements but do not attempt to optimize all the components. In this section, we present the general elements of our theory. In the Section “Computational methods,” we describe the choices of models used in the computer simulations.

We denote a series with beats by their times


(1) $${\overset{⌣}{t}}_{N}=(t_{N},...t_{1}),$$

where a subindex *i* indicates the *i*^th^ beat and *N* is the number of beats in a particular experimental trial. Another notation introduced in Equation 1 is the use of the accented variables ${\overset{⌣}{x}}_{i}$ to indicate the temporal history of the variable *x* up to the moment *i* (Burgi et al., 2000; Grzywacz and De Juan, 2003). Because we use an isochronic condition in our experiments (Figure 1D), the times of the beats are such that


(2) $$\begin{array}{l} t_{i}-t_{i-1}=\Delta ,1<i<N\\ T_{N}-t_{N-1}=\Delta \;(1+\rho ), \end{array}$$

where Δ > 0 and ρ are constants, with the former and latter indicating the typical inter-beat interval and the relative deviation of the last interval respectively. These beats are linked to noisy spike trains in the brain (Figures 1A–C). To simplify our modeling, we assume that we can capture the spike train due to the beat at time *t_i_* with a single instant in the train, *s_i_* > *t*_i_. This instant could represent, for example, the mean or median of the times of the spikes. These instants are denoted here by ${\overset{⌣}{s}}_{N}=(s_{N},...,s_{1})$.

Because of the noise in the spike trains, we assume that the beats are linked to the internal times of the spikes by the probability density distribution


(3) $$P\;(s_{i}|t_{i}:\vec{\alpha })=P_{s}(s_{i}|t_{i}:\vec{\alpha }),$$

where in this article, we use the symbol “:” to separable the variables (for example, *t_i_*) from the vector of parameters ($\vec{\alpha }$ in Equation 3). Because of causality, the function *P_s_* must obey $P_{s}(s_{i}|t_{1}:\vec{\alpha })$) = 0 for *s_i_* < *t_i_*. Moreover, in this article, we postulate that *P_s_* is temporally asymmetric.

Let us assume for simplicity that the brain knows somehow that the first *N* − 1 beats are isochronic (Equation 2). Thus, if the brain estimates the time of the first beat ($t_{1}^{\left(e\right)}$) and the inter-beat interval (Δ^(*e*)^), then the brain automatically has an estimate of the time of any beat up to the penultimate:


(4) $$t_{i}^{\left(e\right)}=t_{1}^{\left(e\right)}+(i-1)\;\Delta^{\left(e\right)},1\leq i\leq N-1.$$

From the estimate of $t_{N-1}^{\left(e\right)}$ and Equation 3, the brain can obtain an optimal prediction for the time of the last spike, $s_{N}^{\left(p\right)}$. The optimal subject would then compare the time of this prediction with that of the actual last spike to respond to the question in the experiment in Figure 1D:


(5) $$s_{N}^{\left(p\right)}\{\begin{array}{c} <\;s_{N}\;subject\;answers\;"Late"\\ >\;s_{N\;}subject\;answers\;"Early" \end{array}.$$

To obtain optimal estimates of $t_{i}^{\left(e\right)},\Delta^{\left(e\right)},s_{N}^{\left(p\right)}$, we need to know the probability $\text{P}\left(t_{1}^{\left(e\right)},\Delta^{\left(e\right)},s_{N}^{\left(p\right)}|{\hat{s}}_{N-1}\right),$, that is, the probability of our desired estimates and prediction given the history of the spikes. Using Bayes theorem (Koop, 2003; Knill and Pouget, 2004; Doya et al., 2007; Robert, 2007), we get


$$\text{P}\left(t_{1}^{\left(e\right)},\Delta^{\left(e\right)},s_{N}^{\left(p\right)}|{\overset{⌢}{s}}_{N-1}\right)=K\text{P }\left({\overset{⌢}{s}}_{N-1},s_{N}^{\left(p\right)}|t_{1}^{\left(e\right)},\Delta^{\left(e\right)}\right)P\left(t_{1}^{\left(e\right)},\Delta^{\left(e\right)}\right),$$

where *K* is a normalization constant independent of $t_{1}^{\left(e\right)}$, Δ^(*e*)^, and $s_{N}^{\left(p\right)}$, the variables that we want to optimize in our calculations. A simplification of this equation is possible by noting that we can drop the dependence on $t_{1}^{\left(e\right)}$ from the second probability function of the right-hand side. We can drop this dependence because no special time for the first beat exists. Consequently, we can rewrite this equation as


(6) $$\text{P}\left(t_{1}^{\left(e\right)},\Delta^{\left(e\right)},s_{N}^{\left(p\right)}|{\overset{⌢}{s}}_{N-1}\right)=K\text{P }\left({\overset{⌢}{s}}_{N-1},s_{N}^{\left(p\right)}|t_{1}^{\left(e\right)},\Delta^{\left(e\right)}\right)P\left(\Delta^{\left(e\right)}\right),$$

The interpretation of this equation follows standard Bayesian concepts. The first probability function is the likelihood function because is predicts the responses of the brain given the beat under consideration (Equation 4). In turn, the second probability function encapsulates the prior assumption about the most common rhythms heard in the world (information about common inter-beat intervals as stated in our Introduction). We denote this later function by ${\text{P}}_{\Delta }(\Delta^{*}:\vec{\beta })$, where its vector of parameters is $\vec{\beta }$.

To find the optima of Equation 6, we propose for simplicity approximating $t_{1}^{\left(e\right)}$, Δ(*e*), and $s_{N}^{\left(p\right)}$ in two stages, as follows:


(7) $$\left(t_{1}^{\left(e\right)}(\vec{\alpha },\vec{\beta }),\Delta^{\left(e\right)}(\vec{\alpha },\vec{\beta })\right)=\arg\max_{t^{*},\Delta^{*}}P_{\Delta }(\Delta^{*}:\vec{\beta })\prod_{i\;=\;1}^{N-1}P_{s}\left(s_{i}|T^{*}+(i-1)\Delta^{*}:\vec{\alpha }\right),$$

(8) $$s_{N}^{\left(p\right)}\left(\vec{\alpha },\vec{\beta },\vec{\gamma }\right)=\arg\max_{s^{*}}\int_{t_{1}^{\left(e\right)}+\;(N-1)\;\Delta^{\left(e\right)}}^{\infty }ds^{'}P_{s}\left(s^{'}|t_{1}^{\left(e\right)}+(N-1)\Delta^{\left(e\right)}\right)L(s^{*},s^{'}:\vec{\gamma }),$$

where the *L* function is the loss function for $s_{N}^{\left(p\right)}$ and $\vec{\gamma }$ is the vector of parameters of this function. We use an explicit loss function for Equation 8 but not for Equation 7 because the task requires only a decision for $s_{N}^{\left(p\right)}$ (Equation 5) but not for $t_{1}^{\left(e\right)}$ and Δ(*e*). The loss function measures the cost of error between s* and *s*^′^.

## Computational Methods

### Particular models used in the simulations

In Section “Theory part 2: computational modeling,” we presented the general elements of our theory. In this section, we describe the choices of models used in computer simulations.

To perform the computer simulations, we must specify three models, namely, the functions $P_{s}(s_{i}|t_{1}:\vec{\alpha })$ (Equation 3), ${\text{P}}_{\Delta }(\Delta^{*}:\vec{\beta })$ (Equation 7), and $L\;(s^{*},s^{'}:\vec{\gamma })$ (Equation 7). In this article, we postulate that *P_s_* is temporally asymmetric. To model this asymmetry, we use the Gamma distribution as commonly employed in neuroscience in its original form or as a normalized alpha function (Grzywacz et al., 1988; Destexhe et al., 1994; Van Vreeswijk et al., 1994):


(9) $$P_{s}\left(s_{i}|t_{i}:\vec{\alpha }=(\tau_{I},n)\right)=\frac{{\left(s_{i}-t_{i}\right)}^{n-1}}{\tau_{I}{}^{n}\Gamma (n)}e^{-\frac{\left(s_{i}-t_{i}\right)}{\tau_{I}}},$$

where *τ_I_* is the time constant associated with the Impulse response from beat to spike, *n* ≥ 1 is the parameter controlling the asymmetry of *P_s_* (this function becomes increasingly symmetric as *n* increases), and Γ is the Gamma function.

Next, because Δ > 0, we model *P*_Δ_ in this article as a lognormal distribution (Krishnamoorthy, 2006) instead of the more standard normal distribution:


(10) $$\text{P}\left(\Delta^{\left(e\right)}:\vec{\beta }\right)={\text{P}}_{\Delta }\left(\Delta^{\left(e\right)}:\vec{\beta }=(\tau_{\Delta },\sigma )\right)=\frac{1}{\frac{\Delta^{\left(e\right)}}{\tau_{\Delta }}\sqrt{2\pi }\sigma }e^{\frac{{\left(\log\left(\frac{\Delta^{\left(e\right)}}{\tau_{\Delta }}\right)\right)}^{2}}{2\sigma^{2}}},$$

where $\vec{\beta }$ is the vector of parameters of the prior distribution. In the model of Equation 10, the components of $\vec{\beta }$ are *τ_Δ_*, a time constant making the variable Δ^(*e*)^*τ*_Δ_ unitless and σ > 0, a constant controlling the symmetry of the distribution. The smaller σ is, the more the distribution becomes symmetrical and normal. Finally, the full lognormal model includes a constant subtracted from the logarithm inside the square. We did not include this constant here because we both did not need it to capture what we wanted to model and did not want to add parameters to the study.

Finally, the loss function $L\;(s^{*},s^{'}:\vec{\gamma })$ can be modeled in different ways and in this article, we explored four types:


(11) $${\text{L}}_{M}\left(s^{*},s^{'}:\vec{\gamma }=\left(\tau_{E},\tau_{S}\right)\right)=\left(1-\frac{\left(s^{*}+\tau_{E}\right)}{\tau_{S}}{\frac{s}{\tau_{S}}}^{'}\right),$$

(12) $${\text{L}}_{\delta }(s^{*},s^{'}:\vec{\gamma }=\tau_{E})=\delta \left((s^{*}+\tau_{E})-s^{'}\right),$$

(13) $${\text{L}}_{2}(s^{*},s^{'}:\vec{\gamma }=\tau_{E})={\left((s^{*}+\tau_{E})-s^{'}\right)}^{2},$$

(14) $${\text{L}}_{1}(s^{*},s^{'}:\vec{\gamma }=\tau_{E})=\;|(s^{*}+\tau_{E})-s^{'}|.$$

The loss function in Equation 11 is based on a standard Margin-based, convex, proper, and classification-calibrated loss function frequently used in binary classification (Rosasco et al., 2004; Bartlett et al., 2006). The function of the τ*_S_* parameter is to make the s* and *s*^′^ terms unitless, so that their multiplication can be subtracted from 1 in Equation 11. In turn, the parameter *τ* ≥ 0 is the simplest implementation of the Better-Early-than-Late loss (BELL). The idea is that the s* term becomes similar to the *s*’ term if the predicted s* happens earlier than in reality by *τ_E_*. A similar principle for the role of applies to the other loss functions. However, these functions emphasize different losses. That in Equation 12 uses the δ of Dirac, which forces a solution optimizing the maximum a posteriori solution (Robert, 2007; Bassett and Deride, 2019). Therefore, the brain would estimate the time of the last spike as the time of the estimated last beat (Equation 4) plus the mode of *P_s_*. In turn, Equations 13 and 14 force the minimization of quadratic and absolute-value errors. These minimizations tend to lead to a predicted time of the last spike that is the sum of the time of the estimated last beat, and the mean and the median of *P_s_* respectively (Koop, 2003).

### Parameters of the simulation

In this article, we report on simulations with different parameter sets of the theoretical models. The models had either five or six free parameters, namely, *τ_I_* and *n* in Equation 9, *τ*_Δ_ and σ in Equation 10, and *τ_E_* and τ*_S_* in Equations 11–14. We optimized the parameters with a coordinate-descent algorithm (Wright, 2015) by minimizing the *χ*^2^ of the goodness of fit. The initial condition of the optimizations was *τ_I_* = 0.1 *s*, *n* = 2, *τ*_Δ_ = 2 *s*, σ = 1, *τ_E_* = 0.01 *s* and τ_S_ = 0.1 *s*. These values of initial conditions were chosen by inspection of neuroscience literature or for the sake of simplicity. The choice of *n* = 2 was because “2” was the smallest integer yielding a rise-and-fall asymmetric impulse response (Krishnamoorthy, 2006). With this choice, a selection of τ*_I_* = 0.1 *s* sets the mode of the impulse-response distribution at 100 ms (Krishnamoorthy, 2006), a reasonable value for auditory cortical impulse responses (Lü et al., 1992; Curio et al., 2000; Norman-Haignere et al., 2022). Next, the choice σ = 1 is the smallest integer compatible with Equation 10 and with this selection, the mode of the distribution happens approximately when $\Delta^{\left(e\right)}/\tau_{\Delta }\approx 1/3.$ Thus, to set the mode of Δ^(*e*)^, in the middle of the experimental range, we made *τ*_Δ_ = 2 s. We then set *τ_E_* = 0.01 *s*, thinking that BELL would make a small correction, namely, only 10 ms. Finally, given that we set the mode of the impulse response at around 100 ms, we also set τ*_S_* to the same scale.

We then designated the optimal set of parameters as our standard set because the corresponding results captured the data reasonably well. We also performed simulations with other parameters around the test set to study the roles of the different parameters of the model. The parameters of each simulation are indicated as appropriate in the Results.

### Computer simulations

The optimization simulations for any given loss function proceeded with the following algorithm:

a. Initialize the optimal theoretical parameter set as indicated in the Section “Parameters of the simulation.”
b. Initialize $\chi_{opt}^{2}$ = 10^300^.
c. Make the current theoretical parameter set equal to the optimal theoretical parameter set.
d. Initialize the parameters of the experiment, namely, *N* = 3, Δ = 0.15 *s*, and ρ = −0.15 (Equations 1 and 2).
e. Obtain the beat times (Equation 2).
f. Repeat the following Steps i-vi 1,000 times.

i. Sample the time of the spikes (Equation 9).
ii. Estimate the time of the first beat and the inter-beat interval (Equation 7).
iii. Estimate the time of the last spike (Equation 8), using the different loss functions (Equations 11–14).
iv. Determine if the estimated time of the last spike was before or after the actual last spike (Equation 5).
v. The response was correct if ρ < 0 and the estimated time of the last spike was after the actual last spike or vice-versa.
vi. Accumulate the number of correct responses.

g. Calculate the theoretical percentage of correct responses by dividing the number of correct responses by 1,000.
h. Calculate the predicted number of correct responses by multiplying the result in Step g by the number of experimental trials per condition.
i. Calculate the contribution to the *χ*^2^ for the current experimental parameter set.
j. Check if we have run over all sets of experimental parameters.

i. If yes

A. Sum all *χ*^2^ from Steps g.
B. If this sum is smaller than $\chi_{opt}^{2}$

1. Reset $\chi_{opt}^{2}$ to the sum.
2. Reset the optimal theoretical parameter set to the current theoretical parameter set.
3. Can we update the current theoretical parameter set of the simulation according to the coordinate-descent algorithm?

I. If yes, make the update and go to Step d.
II. Otherwise, end the procedures.

ii. Otherwise, update the experimental parameter set and go back to Step e.

### Quantification of fits

With the *χ*^2^ statistic, we used a Goodness of Fit test for the null hypothesis that the model fits the data by chance (Krishnamoorthy, 2006). We applied the test to the complete data set encompassing Figure 3 and Figure 5. The degrees of freedom for this test varied across loss functions. This is because they had different numbers of theoretical parameters (Equations 11–14). In general, the number of degrees of freedom was the number of experimental parameters (4 × 4 × 6 = 96) minus the number of theoretical parameters (6 and 5 for Equation 11 and Equations 12–14 respectively) minus 1. Thus, we had 89 degrees of freedom for the first loss function and 90 for the others.

## Computational Results

### Fits of the data

**Figure 7:**
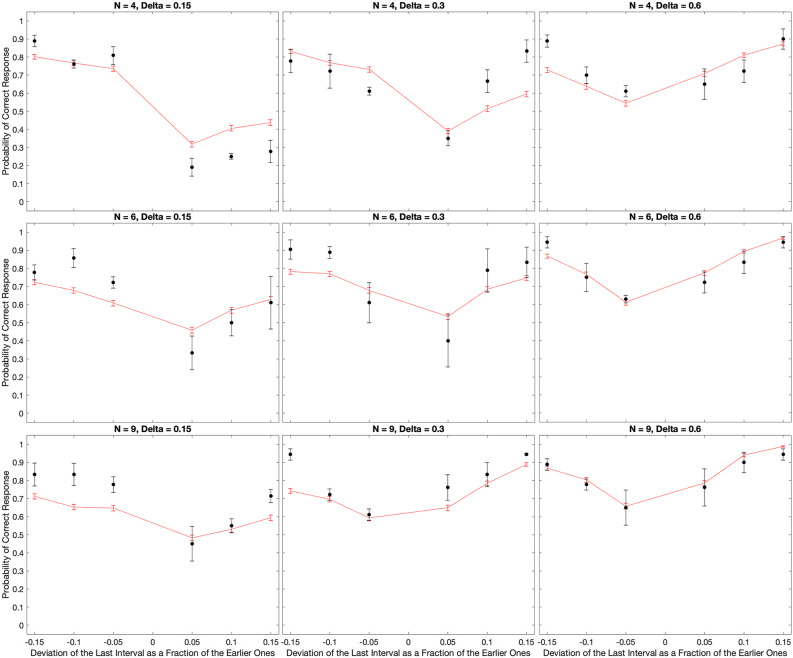
Fits of the model to the median of the data across all subjects. The black dots show the experimental data and their standard errors. In turn, the red lines indicate the behavior of the model with optimal parameters and the absolute-value loss function (Equation 14). With few exceptions, the fits of the model capture well the behavior of the data both quantitatively and qualitatively.

The experimental results confirmed the expectations of systematic errors in the perception of the timing of beats. The results revealed complex behavior as a function of the number of beats and the inter-beat interval (Figures 2–Figure 6). We thus developed a quantitative theory to try to capture these complexities (Equations 5–14). As a first step, we fitted the predictions of a model based on the theory to the median data (3). The results of this fit appear in Figure 7.

Figure 7 reveals that the model captures the complex qualitative features of the experimental data. Not surprisingly, both the data and the model exhibited U-shape behaviors as a function of the relative deviation of the final interval (see Discussion after Figure 3). More interestingly, the model captures the systematic errors observed in the data. When the inter-beat interval was short (0.15 s), both the data and the model reveal the same systematic error: subjects and model too often responded “Early” even when the last beat came late. Thus, the percentage of correct responses was lower than the chance for positive but not negative deviations of the last interval. However, the differential effect of negative vs. positive deviations fell as the number of beats increased. In addition, this difference inverted direction in both the data and the model when the inter-beat interval was longer in duration (for example, 0.6 s). Finally, the inversion also occurred as the number of beats increased at intermediate inter-beat intervals (0.3 s).

The model similarly provides good quantitative fits of the data. This quantitative support can be observed by determining how close the red lines were to the black dots in Figure 7. Confirmation of the good quality of fit arose by a *χ*^2^ test, which showed that the model was statistically indistinguishable from the data. Further confirmation of the success of the model came from its accountability of the biases of the zero-deviation measurements. The model-predicted biases have the same behavior as the experimental one.

However, the fits were not perfect. For example, in Figure 7, the model appeared to overestimate performance for positive deviations of the last interval when *N* = 4 and Δ = 0.15 *s*. In contrast, when *N* = 9 and Δ = 0.15 *s*, the model underestimated performance for negative deviations.

### Varying parameters and loss functions

**Figure 8:**
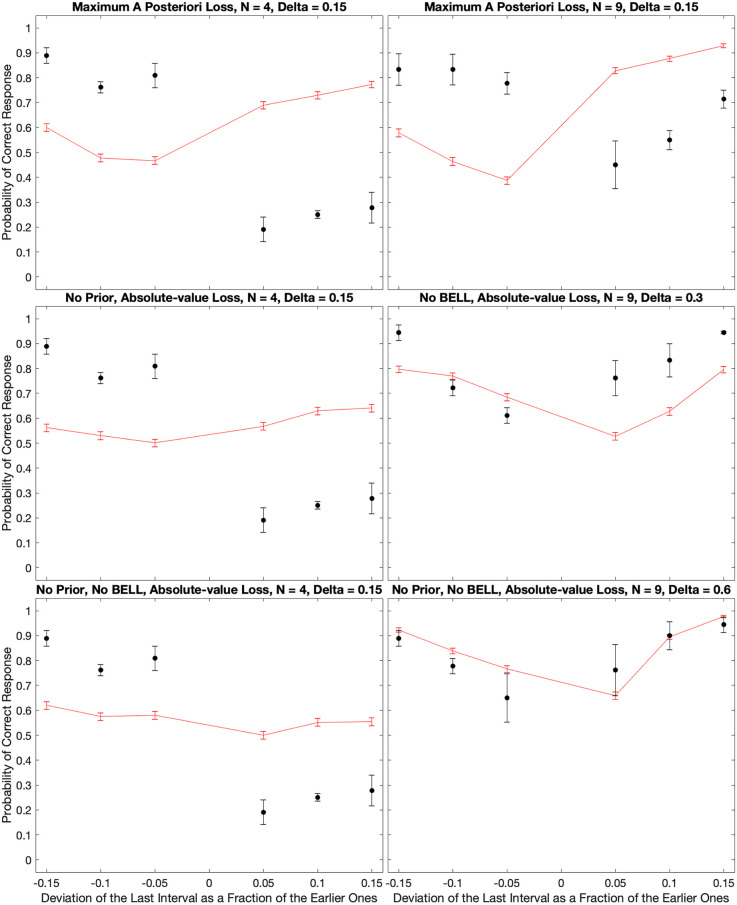
Consequences of relaxing the assumption of the *L*_1_ loss function and making the parameters less optimal. The panels illustrate what happens to the fits in Figure 7 when we use the Maximum-*a-priori* loss function, or when we remove the prior distribution, BELL, or both. With the Maximum-*a-priori* loss, the model cannot account for subjects too often responding “Early” even when the last beat comes late. The same happens when we remove the prior. Without BELL, the effect is more difficult to respond “Late” even when the last beat comes early. Finally, without prior and BELL, the only source of temporal asymmetry is in the impulse response to the beat. Although the effects are smaller, this asymmetry can capture some of both the “Early” and “Late” systematic errors.

In Figure 7, the good fits of the model depended on the choice of the loss function and the optimization of parameters. That figure illustrated the model behavior when using the Absolute-value Loss (L_1_ – Equation 14). In turn, the optimal parameters of the model were *n* = 1, *τ_I_* = 75 *ms*, *τ_E_* = 15 *ms*, *τ_D_* = 1.4 *s*, and σ = 1.1. What were the consequences of relaxing the assumption of the *L*_1_ loss function and making these parameters less optimal? Figure 8 answers these questions with illustrations of further outcomes of the computer simulations.

When we varied the loss function, the fits were almost identically the same for the Margin-based (L*_M_* – Equation 11), Quadratic (L_2_ – Equation 13), and Absolute-value (L_1_ – Equation 14) losses. To emphasize this point, the *χ*^2^ values of the model fits were 56.2, 58.5, and 56.2 for L*_M_*, *L*_2_, and L*_1_* respectively. However, the Maximum-a-posterior loss (L_Δ_ – Equation 12) did much more poorly. The *χ*^2^ value for the best model fit with L_Δ_ was 159.0, almost three times worse than with the other loss functions. An illustration of what went wrong with L_Δ_ appears in Figure 8. As this figure reveals, the model could not account for subjects too often responding “Early” even when the last beat came late for cases of low inter-beat interval.

The variation of the parameters helped determine their roles in the fits of the model. For example, if we removed the prior (Equations 7 and 10) from the equations, the model became incapable of reproducing the too-often-Early bias (compare Figure 8 with Figure 7). This was not surprising because the optimal parameters for prior were τ_D_ = 1.4 *s*, and σ = 1.1. The mean of the prior with these parameters was 2.6 *s*, which was longer than the inter-beat intervals in the experiment. Thus, if anything, the prior caused a bias in the estimations towards late last beats. In contrast, BELL had the opposite effect. Removing BELL by setting *τ_E_* = 0 *ms* instead of 15 *ms* caused some of the too-often-Early bias to disappear (compare Figure 8 with Figure 7. That was also not surprising because, by design, BELL biased the estimations towards earlier times. Finally, we probed the effects of the beat temporally asymmetric impulse response. We did so by removing simultaneously the effects of the prior and BELL. Inspection of Figure 8 revealed that the asymmetric impulse response could by itself produce both too-often-Early and too-often-Late biases. However, the biases are smaller than what we get when incorporating the prior and BELL in the model. In the Discussion, we analyze how the asymmetric impulse response leads by itself to the biases observed in Figure 8.

## Discussion

### The brain does not estimate time correctly

“Not quite my tempo,” retorts J.K. Simmons in the 2014 film, Whiplash, to a training drummer before angrily hurling a chair at the student. Simmons then asks the question, “Were you rushing, or were you dragging?” In other words, Simmons’ character claims a knack for differentiating the fine timing differences within rhythms. However, our study demonstrated that such timing perceptions were not necessarily accurate. In addition, we found that these perceptual inaccuracies were systematic and their direction changed based on the tempo or the individual inter-beat interval durations. Our subjects showed the least probability for these errors with a standard 100 beats per minute stimulus (inter-beat interval of 0.6 s). However, as the rate increased, so too did the probability that the subjects would perceive a “dragging beat.” Thus, at high rates, the beat sounded earlier than expected. In contrast, in line with previous studies, we also demonstrated that when the rate of the stimulus decreased, the probability of a “perceived rushing” increased (Di Luca and Rhodes, 2016; Rhodes, 2018).

### Although incorrect, the times estimated by the brain may be optimal

The relative success of our Bayesian model in capturing the experimental data suggests that the brain has good reasons not to estimate the tempo correctly. The Bayesian nature of our model arises because the impulse response in the brain is noisy. Consequently, under a Bayesian scheme, the best that the brain can hope for is to estimate timing as well as possible. What does “well as possible” mean? If the brain is Bayesian when estimating time, “well as possible” means optimally from a probabilistic perspective (Koop, 2003; Knill and Pouget, 2004; Doya et al., 2007; Robert, 2007). And the computation could be optimal even if the outcome is incorrect. The goal of the brain is not necessarily to estimate time right but to do it in such a way as to optimize survival under natural selection.

Our model suggests three fundamentally different reasons why the brain does not estimate time correctly. First, the prior distribution in the model reflects properties of the external world (Yuille et al., 2004; Geisler and Perry, 2009; Girshick et al., 2011). If the external world has a bias towards certain time intervals, the brain would be better off guessing its estimate to match them, making the outcome sometimes wrong. Second, the temporally asymmetric impulse response and its related transfer function reflect unavoidable biophysical constraints internal to the nervous system (Grzywacz et al., 1992; Destexhe et al., 1994). The optimization achieved by the brain cannot ignore these constraints even if sometimes the estimated time turns out to be wrong. Third, the Better-Early-than-Late Loss function reflects the properties of the action that the animal must perform. Errors in some actions are more costly than others, such as when escaping danger.

The failures of the various alternatives of the model are also instructive. The simplest failure is that the models of this kind that we consider outperform humans if the number of beats is large. That is not surprising because we did not include decision noise in our models (Mueller and Weidemann, 2008; Wilson et al., 2014; Kahneman et al., 2021) and thus, they can improve unbounded with more data. A more interesting type of failure is that of the model with maximum-a-posteriori loss function. This model does not capture the bias towards “Early” responses with short inter-beat intervals (Figure 8). The only way that a loss function affects our results is by interacting with the probabilistic impulse response (Equation 8). The impact of BELL through such a function is relatively small (Figure 8). Hence, the failure of the maximum-*a-posteriori* loss function must have to do with the temporal asymmetry of the impulse response. This function estimates the time of the last spike as follows: it adds to the estimated time of the last beat and the relatively small time-to-peak of the asymmetric impulse response (see discussion after Equation 14). Consequently, the true last spike is almost always after the estimated spike, leading to a “Late” response. Because the data show a bias towards “Early” responses for short inter-beat intervals, the maximum-a-posteriori loss function is unlikely to be what the brain uses.

An alternative beyond those proposed by our model may explain why the systematic time-prediction errors are different for short and long inter-beat intervals. Interval-perception research has suggested that different timescales are tied to different cognitive functions (Buhusi and Meck, 2005). Hence, these timescales would be processed by separate neural networks. If two different networks contribute to short and long timescales, then the errors for short and long inter-beat intervals can be distinct. However, this explanation based on different networks can be compatible with our model. For example, a network implementing the BELL loss function would tend to estimate the next beat as earlier than it occurs. Another network implementing the prior distribution (De Jong et al., 2021) could estimate the next beat as later than it occurs if the inter-beat interval is short.

### Where in the brain is beat time estimated and temporal asymmetries produced?

Beat perception studies between humans and non-human primates indicate that we seem to have evolved uniquely good mechanisms for perceiving time differences (Merchant et al., 2015). Functional imaging and lesion studies have noted the roles of multiple brain areas in carrying out these functions. The areas include the cerebellum, basal ganglia, inferior parietal cortex, prefrontal cortex, premotor cortex, thalamus, and supplementary motor area (Ivry and Spencer, 2004; Kung et al., 2013; Kasdan et al., 2022). In particular, the cerebellum has functional significance in the predictive coding of temporal information in speech (Lesage et al., 2017). Furthermore, studies focused on the perception of beats with only two pulses have highlighted the key role of the cerebellum in absolute timing measurements (Nichelli et al., 1996; Malapani et al., 1998; Grube et al., 2010). In turn, the neural correlates of our proposed temporally asymmetric impulse responses are general in the brain. Multiple mechanisms exhibit such asymmetries, such as sensory transduction (Grzywacz et al., 1992) and synaptic transmission (Bi and Wang, 2002). These mechanisms lead to asymmetries in sensory processing, particularly in the auditory system (Phillips et al., 2002; Deneux et al., 2016).

### Can the motor system correct the estimation of time?

Humans often listen to musical rhythms while entraining some movement to the beat through tapping or head bobbing, for example. Pure beat perception relies upon an accurate bilateral network of motor areas (Grahn and Brett, 2007). This perception may utilize these motor regions for the purposes of action-oriented simulations, as indicated by the ASAP hypothesis (Patel and Iversen, 2014). Moreover, the production of rhythms through sensorimotor synchronization engages the motor cortex properly (Kasdan et al., 2022). The participation of the motor system may give some indication as to how musicians and dancers can predict the timing of external rhythms despite the occurrence of perceptual systematic errors. The motor system may also help accurately carry out movements within the timing of these intervals. Indeed, this motor activation and the subsequent input of somatosensory and proprioceptive information into the cerebellum has been highlighted as a feedback mechanism for error correction in sensorimotor synchronization (Repp, 2005; Zatorre et al., 2007). Similarly, musical expertise improves the efficiency and fluency of these mechanisms (Loehr et al., 2011). Thus, we may be able to support J.K. Simmons’ character’s evaluation of the musical rushing or dragging. We do it by noting the use of his arms to conduct the ensemble with a preferred tempo. Alternatively, he may have simply used relative perception in his evaluations. If all members of a musical ensemble have similar systematic errors, then the dragging or rushing of an individual may be an added mistake to the collective biases.

### Implications for aesthetic values of rhythm

How can people enjoy musical rhythm if the perception of beat times is wrong? We see three possible answers to this question. First, the most popular tempos are in the range of 90–99 beats per minute (Xiao’an, 2021). These tempos correspond to isochronic inter-beat intervals of around 0.63 s. In our data, such intervals are around those yielding the least systematic errors in the estimation of time (Figure 2 and Figure 4B). Second, even if people get the timing of beats too soon or too late, two consecutive beats tend to have similar systematic errors. Hence, the interval between these beats is statistically correct. We predict that people will only stop enjoying the rhythm if the intervals start varying by more than the intended inter-beat intervals yielding at least 75% correct response. These threshold error intervals vary by the number of beats and the inter-beat interval. For the subject in Figure 2; for 9 beats, these threshold relative errors are approximately 20%, 10%, 10%, and ≫20% for inter-beat intervals of 0.15 s, 0.3 s, 0.6 s, and 1.2 s respectively. Third, when an ensemble of musicians is playing together, the key is for them to synchronize their beats. The motor system may have a more important role than the auditory system in this synchronization (Section “Can the motor system correct the estimation of time?”). In addition, even if musicians in an ensemble perceive every beat incorrectly, this would not matter if all players in the ensemble synchronize their errors.

A potentially powerful conclusion from our modeling studies is that the systematic errors that the brain makes when estimating the time of beats may have a link to why humans enjoy rhythms. The modeling suggests that the brain has purposeful mechanisms when estimating time. These mechanisms may not always estimate the time right, but the errors may reflect the optimization of survival goals. Thus, as we Discuss in “Where in the brain is beat time estimated and temporal asymmetries produced?” the brain devotes purposeful resources to the estimation of time. These resources make this estimation fluent. Therefore, according to the Processing Fluency Theory (Reber et al., 2004; Aleem et al., 2017, 2019), the estimation of time with small, albeit systematic, errors should be aesthetically pleasing to the brain. We propose that the brain does not abhor errors, but may even like them if they help us survive.

## Data Availability Statement

The original contributions presented in the study are included in the article, further inquiries can be directed to the corresponding author.

## Ethics Statement

The studies involving human participants were reviewed and approved by Georgetown University Institutional Review Board. The patients/participants provided their written informed consent to participate in this study.

## Author Contributions

JM designed the experiment, developed its computer code, and analyzed the data. He also wrote half of the first draft of the article and worked on later drafts. HA helped with the development of the experimental computer code and data analysis, and worked on later drafts of the article. NG helped with the experimental design and the data analysis, developed the theoretical framework, carried out the computer simulations, and performed the statistical analysis of their results. He also wrote half of the first draft of the manuscript and worked on later drafts. All authors contributed to the article and approved the submitted version.

## Funding

The research was supported by funds provided by the Provost of Georgetown University and the Board of Trustees of Loyola University Chicago.

## Conflict of Interest

The authors declare that the research was conducted in the absence of any commercial or financial relationships that could be construed as a potential conflict of interest.

## Publisher’s Note

All claims expressed in this article are solely those of the authors and do not necessarily represent those of their affiliated organizations, or those of the publisher, the editors and the reviewers. Any product that may be evaluated in this article, or claim that may be made by its manufacturer, is not guaranteed or endorsed by the publisher.

## References

[B1] Ahissar M. Hochstein S. (2004). The reverse hierarchy theory of visual perceptual learning. Trends Cogn. Sci. 8, 457–464. 10.1016/j.tics.2004.08.011 15450510

[B2] Aleem H. Correa-Herran I. Grzywacz N. M. (2017). Inferring master painters’ esthetic biases from the statistics of portraits. Front. Hum. Neurosci. 11:94. 10.3389/fnhum.2017.00094 28337133 PMC5343217

[B3] Aleem H. Pombo M. Correa-Herran I. Grzywacz N. M. (2019). “Is beauty in the eye of the beholder or an objective truth? A neuroscientific answer,” in Mobile Brain-Body Imaging and the Neuroscience of Art, Innovation and Creativity, eds Contreras-Vidal J. Robleto D. Cruz-Garza J. G. Azorin J. M. Nam C. S. (Cham: Springer International Publishing), 101–110.

[B4] Allefeld C. Görgen K. Haynes J.-D. (2016). Valid population inference for information-based imaging: from the second-level t-test to prevalence inference. Neuroimage 141, 378–392. 10.1016/j.neuroimage.2016.07.040 27450073

[B5] Barraza J. F. Grzywacz N. M. (2008). Speed adaptation as Kalman filtering. Vis. Res. 48, 2485–2491. 10.1016/j.visres.2008.08.011 18782586

[B6] Bartlett P. L. Jordan M. I. Mcauliffe J. D. (2006). Convexity, classification and risk bounds. J. Am. Stat. Assoc. 101, 138–156. 10.1198/016214505000000907

[B7] Bassett R. Deride J. (2019). Maximum a posteriori estimators as a limit of Bayes estimators. Math. Program. 174, 129–144. 10.1007/s10107-018-1241-0

[B8] Bengtsson S. L. Ullen F. Ehrsson H. H. Hashimoto T. Kito T. Naito E.. (2009). Listening to rhythms activates motor and premotor cortices. Cortex 45, 62–71. 10.1016/j.cortex.2008.07.002 19041965

[B9] Bi G. Q. Wang H. X. (2002). Temporal asymmetry in spike timing-dependent synaptic plasticity. Physiol. Behav. 77, 551–555. 10.1016/s0031-9384(02)00933-2 12526998

[B10] Buhusi C. V. Meck W. H. (2005). What makes us tick? Functional and neural mechanisms of interval timing. Nat. Rev. Neurosci. 6, 755–765. 10.1038/nrn1764 16163383

[B11] Burgi P. Y. Yuille A. L. Grzywacz N. M. (2000). Probabilistic motion estimation based on temporal coherence. Neural Comput. 12, 1839–1867. 10.1162/089976600300015169 10953241

[B12] Button K. S. Ioannidis J. Mokrysz C. Nosek B. A. Flint J. Robinson E. S.. (2013). Power failure: why small sample size undermines the reliability of neuroscience. Nat. Rev. Neurosci. 14, 365–376. 10.1038/nrn3475 23571845

[B13] Choi J.-S. Kim J. J. (2010). Amygdala regulates risk of predation in rats foraging in a dynamic fear environment. Proc. Natl. Acad. Sci. U S A 107, 21773–21777. 10.1073/pnas.1010079108 21115817 PMC3003044

[B14] Cohen J. (2013). Statistical Power Analysis for the Behavioral Sciences. New York, NY: Routledge.

[B15] Cooper W. Blumstein D. T. (2015). “Escape behavior: importance, scope and variables,” in Escaping From Predators: An Integrative View of Escape Decisions, eds Cooper D. Jr. Blumstein W. (Cambridge: Cambridge University Press), 3–14.

[B16] Correll J. Mellinger C. Mcclelland G. H. Judd C. M. (2020). Avoid Cohen’s ‘small’, ‘medium’ and ‘large’ for power analysis. Trends Cogn. Sci. 24, 200–207. 10.1016/j.tics.2019.12.009 31954629

[B17] Curio G. Neuloh G. Numminen J. Jousmäki V. Hari R. (2000). Speaking modifies voice-evoked activity in the human auditory cortex. Hum. Brain Mapp. 9, 183–191. 10.1002/(sici)1097-0193(200004)9:4<183::aid-hbm1>3.0.co;2-z 10770228 PMC6871984

[B18] De Boer E. De Jongh H. (1978). On cochlear encoding: potentialities and limitations of the reverse-correlation technique. J. Acoust. Soc. Am. 63, 115–135. 10.1121/1.381704 632404

[B19] De Jong J. Akyürek E. G. Van Rijn H. (2021). A common dynamic prior for time in duration discrimination. Psychon. Bull. Rev. 28, 1183–1190. 10.3758/s13423-021-01887-z 33661470 PMC8367937

[B20] Deneux T. Kempf A. Daret A. Ponsot E. Bathellier B. (2016). Temporal asymmetries in auditory coding and perception reflect multi-layered nonlinearities. Nat. Commun. 7:12682. 10.1038/ncomms12682 27580932 PMC5025791

[B21] Destexhe A. Mainen Z. Sejnowski T. (1994). An efficient method for computing synaptic conductances based on a kinetic model of receptor binding. Neural Comput. 6, 14–18. 10.1162/neco.1994.6.1.14

[B22] Di Luca M. Rhodes D. (2016). Optimal perceived timing: integrating sensory information with dynamically updated expectations. Sci. Rep. 6:28563. 10.1038/srep28563 27385184 PMC4935895

[B23] Donhauser P. W. Florin E. Baillet S. (2018). Imaging of neural oscillations with embedded inferential and group prevalence statistics. PLoS Comput. Biol. 14:e1005990. 10.1371/journal.pcbi.1005990 29408902 PMC5815621

[B24] Doya K. Ishii S. Pouget A. Rao R. P. (2007). Bayesian Brain: Probabilistic Approaches to Neural Coding. Cambridge, MA: MIT Press.

[B26] Ferster D. (1996). Is neural noise just a nuisance? Science 273:1812. 10.1126/science.273.5283.1812 8815544

[B27] Geisler W. S. Perry J. S. (2009). Contour statistics in natural images: grouping across occlusions. Vis. Neurosci. 26, 109–121. 10.1017/S0952523808080875 19216819 PMC2660385

[B28] Girshick A. R. Landy M. S. Simoncelli E. P. (2011). Cardinal rules: visual orientation perception reflects knowledge of environmental statistics. Nat. Neurosci. 14, 926–932. 10.1038/nn.2831 21642976 PMC3125404

[B29] Grahn J. A. (2012). Neural mechanisms of rhythm perception: current findings and future perspectives. Top. Cogn. Sci. 4, 585–606. 10.1111/j.1756-8765.2012.01213.x 22811317

[B30] Grahn J. A. Brett M. (2007). Rhythm and beat perception in motor areas of the brain. J. Cogn. Neurosci. 19, 893–906. 10.1162/jocn.2007.19.5.893 17488212

[B31] Grube M. Cooper F. E. Chinnery P. F. Griffiths T. D. (2010). Dissociation of duration-based and beat-based auditory timing in cerebellar degeneration. Proc. Natl. Acad. Sci. U S A 107, 11597–11601. 10.1073/pnas.0910473107 20534501 PMC2895141

[B32] Grzywacz N. M. De Juan J. (2003). Sensory adaptation as Kalman filtering: theory and illustration with contrast adaptation. Network 14, 465–482. 10.1088/0954-898X_14_3_305 12938767

[B33] Grzywacz N. M. Hillman P. Knight B. W. (1988). The quantal source of area supralinearity of flash responses in Limulus photoreceptors. J. Gen. Physiol. 91, 659–684. 10.1085/jgp.91.5.659 3418317 PMC2216154

[B34] Grzywacz N. M. Hillman P. Knight B. W. (1992). Response transfer functions of Limulus ventral photoreceptors: interpretation in terms of transduction mechanisms. Biol. Cybern. 66, 429–435. 10.1007/BF00197723 1562647

[B35] Hardy N. F. Buonomano D. V. (2016). Neurocomputational models of interval and pattern timing. Curr. Opin. Behav. Sci. 8, 250–257. 10.1016/j.cobeha.2016.01.012 27790629 PMC5077164

[B36] Heil P. Peterson A. J. (2015). Basic response properties of auditory nerve fibers: a review. Cell Tissue Res. 361, 129–158. 10.1007/s00441-015-2177-9 25920587

[B37] Hellström Å. (1985). The time-order error and its relatives: mirrors of cognitive processes in comparing. Psychol. Bull. 97, 35–61. 10.1037/0033-2909.97.1.35

[B38] Ince R. A. Kay J. W. Schyns P. G. (2022). Within-participant statistics for cognitive science. Trends Cogn. Sci. 26, 626–630. 10.1016/j.tics.2022.05.008 35710894 PMC9586881

[B39] Ivry R. B. Spencer R. M. (2004). Evaluating the role of the cerebellum in temporal processing: beware of the null hypothesis. Brain 127:E13. 10.1093/brain/awh226 15277307

[B40] Jackson J. C. Jong J. Bilkey D. Whitehouse H. Zollmann S. Mcnaughton C.. (2018). Synchrony and physiological arousal increase cohesion and cooperation in large naturalistic groups. Sci. Rep. 8:127. 10.1038/s41598-017-18023-4 29317675 PMC5760525

[B41] Kahneman D. Sibony O. Sunstein C. R. (2021). Noise: A Flaw in Human Judgment. New York, NY: Little, Brown.

[B42] Karmarkar U. R. Buonomano D. V. (2007). Timing in the absence of clocks: encoding time in neural network states. Neuron 53, 427–438. 10.1016/j.neuron.2007.01.006 17270738 PMC1857310

[B43] Kasdan A. V. Burgess A. N. Pizzagalli F. Scartozzi A. Chern A. Kotz S. A.. (2022). Identifying a brain network for musical rhythm: a functional neuroimaging meta-analysis and systematic review. Neurosci. Biobehav. Rev. 136:104588. 10.1016/j.neubiorev.2022.104588 35259422 PMC9195154

[B44] Knill D. C. Pouget A. (2004). The Bayesian brain: the role of uncertainty in neural coding and computation. Trends Neurosci. 27, 712–719. 10.1016/j.tins.2004.10.007 15541511

[B45] Kohler K. J. (2009). Rhythm in speech and language. Phonetica 66, 29–45. 10.1159/000208929 19390229

[B46] Konvalinka I. Xygalatas D. Bulbulia J. Schjødt U. Jegindø E.-M. Wallot S.. (2011). Synchronized arousal between performers and related spectators in a fire-walking ritual. Proc. Natl. Acad. Sci. U S A 108, 8514–8519. 10.1073/pnas.1016955108 21536887 PMC3100954

[B47] Koop G. (2003). Bayesian Econometrics. Hoboken, NJ: John Wiley & Sons.

[B48] Kotz S. A. Ravignani A. Fitch W. T. (2018). The evolution of rhythm processing. Trends Cogn. Sci. 22, 896–910. 10.1016/j.tics.2018.08.002 30266149

[B49] Kramer D. L. Bonenfant M. (1997). Direction of predator approach and the decision to flee to a refuge. Anim. Behav. 54, 289–295. 10.1006/anbe.1996.0360 9268459

[B50] Krishnamoorthy K. (2006). Handbook of Statistical Distributions With Applications. New York, NY: Chapman and Hall/CRC.

[B51] Kung S. J. Chen J. L. Zatorre R. J. Penhune V. B. (2013). Interacting cortical and basal ganglia networks underlying finding and tapping to the musical beat. J. Cogn. Neurosci. 25, 401–420. 10.1162/jocn_a_00325 23163420

[B52] Lesage E. Hansen P. C. Miall R. C. (2017). Right lateral cerebellum represents linguistic predictability. J. Neurosci. 37, 6231–6241. 10.1523/JNEUROSCI.3203-16.2017 28546307 PMC5490062

[B53] Liu E. H. Mercado Iii E. Church B. A. Orduña I. (2008). The easy-to-hard effect in human (*Homo sapiens*) and rat (*Rattus norvegicus*) auditory identification. J. Comp. Psychol. 122, 132–145. 10.1037/0735-7036.122.2.132 18489229 PMC2664539

[B54] Loehr J. D. Large E. W. Palmer C. (2011). Temporal coordination and adaptation to rate change in music performance. J. Exp. Psychol. Hum. Percept. Perform. 37, 1292–1309. 10.1037/a0023102 21553990

[B55] London J. (2012). Hearing in Time: Psychological Aspects of Musical Meter. New York, NY: Oxford University Press. 10.1093/acprof:oso/9780195160819.001.0001

[B56] Lü Z.-L. Williamson S. J. Kaufman L. (1992). Human auditory primary and association cortex have differing lifetimes for activation traces. Brain Res. 572, 236–241. 10.1016/j.jogc.2022.08.012 1611518

[B57] Mackintosh N. J. (2009). Varieties of perceptual learning. Learn. Behav. 37, 119–125. 10.3758/LB.37.2.119 19380888

[B58] Malapani C. Dubois B. Rancurel G. Gibbon J. (1998). Cerebellar dysfunctions of temporal processing in the seconds range in humans. Neuroreport 9, 3907–3912. 10.1097/00001756-199812010-00026 9875727

[B59] Merchant H. Grahn J. Trainor L. Rohrmeier M. Fitch W. T. (2015). Finding the beat: a neural perspective across humans and non-human primates. Philos. Trans. R. Soc. Lond. B Biol. Sci. 370:20140093. 10.1098/rstb.2014.0093 25646516 PMC4321134

[B60] Miller K. D. Troyer T. W. (2002). Neural noise can explain expansive, power-law nonlinearities in neural response functions. J. Neurophysiol. 87, 653–659. 10.1152/jn.00425.2001 11826034

[B61] Mogan R. Fischer R. Bulbulia J. A. (2017). To be in synchrony or not? A meta-analysis of synchrony’s effects on behavior, perception, cognition, and affect. J. Exp. Soc. Psychol. 72, 13–20. 10.1016/j.jesp.2017.03.009

[B62] Montagu J. (2017). How music and instruments began: a brief overview of the origin and entire development of music, from its earliest stages. Front. Sociol. 2:8. 10.3389/fsoc.2017.00008

[B63] Mueller S. T. Weidemann C. T. (2008). Decision noise: an explanation for observed violations of signal detection theory. Psychon. Bull. Rev. 15, 465–494. 10.3758/pbr.15.3.465 18567246

[B64] Nazzi T. Bertoncini J. Mehler J. (1998). Language discrimination by newborns: toward an understanding of the role of rhythm. J. Exp. Psychol. Hum. Percept. Perform. 24, 756–766. 10.1037//0096-1523.24.3.756 9627414

[B65] Nichelli P. Alway D. Grafman J. (1996). Perceptual timing in cerebellar degeneration. Neuropsychologia 34, 863–871. 10.1016/0028-3932(96)00001-2 8822733

[B66] Norman-Haignere S. V. Long L. K. Devinsky O. Doyle W. Irobunda I. Merricks E. M.. (2022). Multiscale temporal integration organizes hierarchical computation in human auditory cortex. Nat. Hum. Behav. 6, 455–469. 10.1038/s41562-021-01261-y 35145280 PMC8957490

[B67] Nozaradan S. (2014). Exploring how musical rhythm entrains brain activity with electroencephalogram frequency-tagging. Philos. Trans. R. Soc. Lond. B Biol. Sci. 369:20130393. 10.1098/rstb.2013.0393 25385771 PMC4240960

[B68] Patel A. D. Iversen J. R. (2014). The evolutionary neuroscience of musical beat perception: the action simulation for auditory prediction (ASAP) hypothesis. Front. Syst. Neurosci. 8:57. 10.3389/fnsys.2014.00057 24860439 PMC4026735

[B660] Peirce J. W. Gray J. R. Simpson S. MacAskill M. Höchenberger R. Sogo H.. (2019). PsychoPy2: experiments in behavior made easy. Behav. Res. Methods 51, 195–203. 10.3758/s13428-018-01193-y 30734206 PMC6420413

[B69] Phillips D. P. Hall S. E. Boehnke S. E. (2002). Central auditory onset responses and temporal asymmetries in auditory perception. Hear. Res. 167, 192–205. 10.1016/s0378-5955(02)00393-3 12117542

[B70] Ramus F. Nespor M. Mehler J. (1999). Correlates of linguistic rhythm in the speech signal. Cognition 73, 265–292. 10.1016/s0010-0277(99)00058-x 10585517

[B71] Reber R. Schwarz N. Winkielman P. (2004). Processing fluency and aesthetic pleasure: is beauty in the perceiver’s processing experience? Personal. Soc. Psychol. Rev 8, 364–382. 10.1207/s15327957pspr0804_3 15582859

[B72] Recio-Spinoso A. Temchin A. N. Van Dijk P. Fan Y.-H. Ruggero M. A. (2005). Wiener-kernel analysis of responses to noise of chinchilla auditory-nerve fibers. J. Neurophysiol. 93, 3615–3634. 10.1152/jn.00882.2004 15659532

[B73] Repp B. H. (2005). Sensorimotor synchronization: a review of the tapping literature. Psychon. Bull. Rev. 12, 969–992. 10.3758/bf03206433 16615317

[B74] Rhodes D. (2018). On the distinction between perceived duration and event timing: towards a unified model of time perception. Timing Time Percept. 6, 90–123. 10.1163/22134468-20181132

[B75] Robert C. P. (2007). The Bayesian Choice: From Decision-Theoretic Foundations to Computational Implementation. New York, NY: Springer.

[B76] Rokem A. Watzl S. Gollisch T. Stemmler M. Herz A. V. Samengo I. (2006). Spike-timing precision underlies the coding efficiency of auditory receptor neurons. J. Neurophysiol. 95, 2541–2552. 10.1152/jn.00891.2005 16354733

[B77] Rosasco L. De Vito E. Caponnetto A. Piana M. Verri A. (2004). Are loss functions all the same? Neural Comput. 16, 1063–1076. 10.1162/089976604773135104 15070510

[B78] Sadibolova R. Sun S. Terhune D. B. (2021). Using adaptive psychophysics to identify the neural network reset time in subsecond interval timing. Exp. Brain Res. 239, 3565–3572. 10.1007/s00221-021-06227-0 34581840 PMC8599254

[B79] Smith E. C. Lewicki M. S. (2006). Efficient auditory coding. Nature 439, 978–982. 10.1038/nature04485 16495999

[B80] Stanley T. D. Carter E. C. Doucouliagos H. (2018). What meta-analyses reveal about the replicability of psychological research. Psychol. Bull. 144, 1325–1346. 10.1037/bul0000169 30321017

[B81] Teich M. C. (1989). Fractal character of the auditory neural spike train. IEEE Trans. Biomed. Eng. 36, 150–160. 10.1109/10.16460 2921061

[B82] Teich M. C. Khanna S. M. (1985). Pulse-number distribution for the neural spike train in the cat’s auditory nerve. J. Acoust. Soc. Am. 77, 1110–1128. 10.1121/1.392176 3980865

[B83] Treder M. S. Purwins H. Miklody D. Sturm I. Blankertz B. (2014). Decoding auditory attention to instruments in polyphonic music using single-trial EEG classification. J. Neural Eng. 11:026009. 10.1088/1741-2560/11/2/026009 24608228

[B84] Van Vreeswijk C. Abbott L. Bard Ermentrout G. (1994). When inhibition not excitation synchronizes neural firing. J. Comput. Neurosci. 1, 313–321. 10.1007/BF00961879 8792237

[B85] Wilson M. Cook P. F. (2016). Rhythmic entrainment: why humans want to, fireflies can’t help it, pet birds try and sea lions have to be bribed. Psychon. Bull. Rev. 23, 1647–1659. 10.3758/s13423-016-1013-x 26920589

[B86] Wilson R. C. Geana A. White J. M. Ludvig E. A. Cohen J. D. (2014). Humans use directed and random exploration to solve the explore-exploit dilemma. J. Exp. Psychol. Gen. 143:2074. 10.1037/a0038199 25347535 PMC5635655

[B87] Wright S. J. (2015). Coordinate descent algorithms. Mathemat. Program. 151, 3–34. 10.48550/arXiv.1502.04759

[B88] Xiao’an L. (2021). Which Musical Tempos are People Streaming the Most? Available online at: https://blog.musiio.com/2021/08/19/which-musical-tempos-are-people-streaming-the-most/

[B25] Yuille A. L. Fang F. Schrater P. Kersten D. (2004). “Human and ideal observers for detecting image curves,” in Advances in Neural Information Processing Systems, Vol. 16, eds S. Thrun, L. Saul, and B. Schölkopf (Cambridge, MA: MIT Press), 59–70.

[B89] Zatorre R. J. Chen J. L. Penhune V. B. (2007). When the brain plays music: auditory-motor interactions in music perception and production. Nat. Rev. Neurosci. 8, 547–558. 10.1038/nrn2152 17585307

